# Utilization of an Intraoperative Grading Scale in Laparoscopic Cholecystectomy: A Nepalese Perspective

**DOI:** 10.1155/2020/8954572

**Published:** 2020-11-24

**Authors:** Suman Baral, Raj Kumar Chhetri, Neeraj Thapa

**Affiliations:** Department of Surgery, Lumbini Medical College and Teaching Hospital Ltd., Pravas, Tansen-7, Palpa, Nepal

## Abstract

**Introduction:**

Difficult geographic diversity and late presentation to medical attention often make the laparoscopic cholecystectomy difficult and chances of conversion and complication remains. Various preoperative grading scales have been developed for predicting the difficulty of surgery in cholecystitis patients; however, intraoperative assessment of anatomical status and inflammation of the gall bladder has not been assessed till date except for some guidelines like the Parkland grading scale (PGS). We aimed to utilise this guideline in patients undergoing laparoscopic cholecystectomy in rural community of the developing nation.

**Methods:**

PGS was applied for all the patients undergoing laparoscopic cholecystectomy and laboratory and outcome factors like preoperative white blood cells (WBC), open conversion, subtotal cholecystectomy, length of surgery, and bile leaks postoperatively were assessed.

**Results:**

Among 178 patients who underwent cholecystectomy, there were 40 grade one GBs, 90 grade two GBs, 26 grade three GBs, 16 grade four GBs, and six grade five GBs. With a conversion rate of 6.74%, eight patients underwent subtotal cholecystectomy. Among them, four patients were graded as 5th grade, two as 4^th^ grade, and two as 3^rd^ grade according to PGS system. Postoperative bile leak was seen in three patients among which two were grade five GBs and one was grade four. Preoperative WBC, conversion to open, subtotal cholecystectomy, length of surgery, and postoperative bile leak all significantly increased with increasing grades (*p* < 0.05).

**Conclusion:**

PGS can be applied in patients undergoing laparoscopic cholecystectomy in the rural setting of a developing nation. With its application, postoperative course could be predicted and adequate counselling can be done about the possibilities of the outcome.

## 1. Introduction

Laparoscopic cholecystectomy is one of the most common surgical procedures performed globally and is the treatment of choice for the management of symptomatic gall stone diseases [1]. Owing to the difficult geographic diversity of the rural population who seek health care at our institute and lack of knowledge of the disease process, late presentation for medical attention often leads to difficulty in surgery with increased risk of complications and conversion. Various preoperative grading scales have been developed for predicting difficulty of surgery in patients undergoing cholecystectomy for cholecystitis which solely depends upon clinical aspects and diagnostic modalities that mainly depict the intraoperative and postoperative outcomes. However, these grading scales have not been validated in a developing country like Nepal probably due to its complexity and ambiguity of anatomical, clinical, and histological aspects. Similarly, intraoperative assessment of anatomical status and inflammation of the gall bladder has not been well studied till date except for few guidelines like the Parkland grading scale (PGS).

PGS is a five-tiered easily implementable grading system based on anatomy and inflammatory changes in the gall bladder due to persistent inflammatory processes that ensue when diseased [2]. We aimed to utilise this guideline and apply it to the patients undergoing laparoscopic cholecystectomy in a low-resource setting at the rural community of the developing nation. We tend to believe that such grading system if applied in daily practises helps us to predict the possible outcome of the surgery, counsel the attendings, and possibility of the early conversion keeping a low level of perseverance while on laparoscopic procedures.

## 2. Methods

This was a prospective cross-sectional study conducted over a period of six months from June 2019 to November 2019 in a tertiary hospital of western Nepal which includes all the patients undergoing laparoscopic cholecystectomy for symptomatic cholelithiasis. Ethical clearance was taken from Institutional Review Committee (Ref No: IRC-LMC 05-G/019). PGS was applied for all the patients, and outcome factors like preoperative white blood cells count, open conversion, subtotal cholecystectomy, length of surgery, and postoperative bile leaks were assessed. It was assumed that the above variables would increase as the severity of the disease increases that could be delineated by an increase in the severity grade. Four faculty surgeons were responsible for the surgery with minimum experience of performing more than 500 laparoscopic cholecystectomies while the PGS classification was done by the author himself in most of the cases wherever feasible. All the surgeries were performed electively whilst acute cases were performed early morning the next day with a maximum wait of around 12 hours as laparoscopic facilities were not available during night times. Intraoperative cholangiogram was not done in any of the cases. PGS is a five-tiered grading system developed by Parkland Memorial Hospital, Texas, USA, in 2017 which is based on a concept of having (1) a limited number of grades, (2) being easy to remember, and (3) having consistent assignment among users applying the anatomical and inflammatory aspects of the disease process [2]. The PGS cholecystitis grade is as shown in Table 1 [3].

The laparoscopic cholecystectomy done at our institute has been a four-port surgery with umbilical port serving for camera, epigastric port, and two lateral 5 mm ports. The Hasson open technique was used to create the umbilical port, and all the patients were assigned as per PGS by the operating surgeon himself. For easily visualized gall bladder, initial view was considered the screen image which was seen once the gall bladder was grasped and retracted cephalad. In cases with severe inflammation and nonvisualized gall bladder, the initial view was described as the view of the inflamed area. A special pro forma was designed including PGS cholecystitis severity criteria and filled out by the operating surgeon himself. The pro forma included peri- and postoperative variables like age of the patient, sex, subtotal cholecystectomy, open conversion, complications like postoperative bile leak within two months, and wound infections. SPSS version 16 was used for statistical analysis, and a *p* value less than 0.05 was considered significant. The Welch's one-way ANOVA was used to evaluate the relationship of the PGS for cholecystitis with the continuous outcomes of length of hospital stay while the Tukey–Kramer test was applied for all pairwise comparison of means between the grades.

## 3. Results

A total of 178 patients were enrolled in the study among which 146 were females and 32 were males. Mean age of female was 44.41 ± 15.47 years while males were 51.13 ± 12.72 years of age. There were 40 grade 1 GBs, 90 grade 2 GBs, 26 grade 3 GBs, 16 grade 4 GBs, and 6 grade 5 GBs. Mean level of ALT was 30.06 ± 32.82 units/L, while mean AST was 29.72 ± 25.50 units/L. Mean level of hemoglobin was 12.52 ± 1.19 g/dL. Gall bladder wall thickness was highest in grade 5 PGS, while pericholecystic collection was seen in only 10 patients in ultrasonography. (Table 2) Similarly, there were 13 patients with type 2 diabetes mellitus among which conversion occurred in 3 of them. Six patients had past history of upper abdominal surgery, and 4 cases had laparoscopy converted to open cholecystectomy.

Table 3 shows the association of perioperative gall bladder grade with other surgical parameters like subtotal cholecystectomy, conversion, postoperative bile leak, and preoperative white cell count. Twelve patients underwent open conversion with a conversion rate of 6.74%, while eight patients underwent subtotal cholecystectomy. Among them, four patients were graded as fifth grade, two as 4^th^ grade, and two as 3^rd^ grade according to the PGS system. Postoperative bile leak was seen in three patients among which two were grade five GBs and one was grade four. Preoperative white cell count increased with increasing Parkland grades. Subtotal cholecystectomy, conversion to open, postoperative bile leak, and preoperative WBC all significantly increased with increasing grades.

Similarly, the Welch one-way ANOVA was used to evaluate the relation between the Parkland grading scale for cholecystitis and continuous outcome of length of surgery which was statistically significant (*p* = 0.0001, Df = 4, and *F* = 6.937). The Tukey–Kramer test for all pairwise comparisons (*p* < 0.05) revealed Grade 1 significantly different from Grade 5, Grade 2 from 5, Grade 3 from 5, Grade 4 from 5, and Grade 5 from 1, 2, 3, and 4. Histological evaluation revealed 150 GBs to have chronic cholecystitis, 15 cases of acute cholecystitis which included 8 grade 2 GBs and 7 grade 3 GBs. Eight specimens turned to have acute on chronic cholecystitis while 3 patients had gangrenous cholecystitis who fall into grade 5 according to PGS (*p* = 0.0001).

## 4. Discussion

Laparoscopic cholecystectomy is one of the most common surgical procedures performed worldwide [1]. Though, laparoscopic cholecystectomy seems to be one of the easiest procedures especially for the beginner surgeons, yet it is equally risky and difficult even for the finest surgeons with more experienced years. Chances of iatrogenic bile duct injury with a significant impact on patient's quality of life have been one of the dreadful situations for the experienced surgeons too, and necessary measures ought to be implemented decreasing the level of perseverance and early conversion wherever deemed necessary [4]. Anatomical variability and inflammatory factors play a key role, and these account for conversion and outcomes of the surgery [2]. Difficult geographical diversity and lack of knowledge about the disease process render the late presentation of patients to seek medical attention, and by the time, they are noticed by the treating surgeon, the golden hour for treatment has already passed, and the course of the treatment happens to change as experienced by the author himself.

Various preoperative grading risk factor criteria have been established and validated till date, and only few such scales like Randhawa et al. and others have taken the intraoperative factors into consideration [5–11]. However, such grading systems have not still gained space for daily clinical activities due to their complexity and difficulty in remembering owing to maximum number of variables which do not allow for effective outcome comparisons [3]. Also, it seems logical that intraoperative scales consider more meaningful comparison of outcomes rather than preoperative grades with which instant decision could be ascertained intraoperatively like conversion or takeover of the surgery by an experienced surgeon that mitigates the potential risk of complications [12]. We tried to consider the parameters like anatomical variation and inflammatory factors that seem responsible for the outcomes in laparoscopic cholecystectomy at our setting in patients with preoperative diagnosis of acute biliary colic and calculus cholecystitis grading the intraoperative findings on the basis of PGS. It includes five-tiered cholecystitis grade considering adhesions, abnormal liver anatomy, stone impaction (Mirrizi), gall bladder perforation, and necrosis and the severity increases as grades increase with the above considered variables [2]. Considering our findings and comparing to other validation studies conducted by the proposers of the PGS, we saw a significant increase in subtotal cholecystectomies, conversion to open, postoperative bile leaks, and increase in preoperative white blood cell. Similarly, Madni et al. in his prospective validation demonstrated an increase in the time duration of surgery along with difficulty of surgery score and postoperative hospital stay duration which corroborated our findings [3].

American Association for the Surgery of Trauma (AAST) had established a grading scale which gives 5 graded definitions for four categories that includes clinical, imaging, operative, and pathological diagnosis of the disease which requires extensive data collection and histology reports take days to be ascertained [13]. In contrast, PGS seems quite easy to determine while in the operating room. Similarly, AAST excludes the other causes of biliary diseases like biliary colic and symptomatic cholelithiasis determining the grades only for acute cholecystitis [3]. Gall bladder wall thickness along with histological evidence of gangrenous cholecystitis could be predicted for higher grades, and this can be attributed to our findings whilst similar findings were demonstrated in a study to develop a cholecystitis stratification system with grades based on intraoperative images by Madni and colleagues [2]. A scale termed as G10 score developed in 2015, which is a 10-point gall bladder operative scoring system was modified recently removing total time to dissect out the critical view, included ten variables with parameters like gall bladder's operative appearance, whether distended or contracted, ease of access, and the presence of sepsis in the peritoneal cavity, either biliary peritonitis or purulent fluid, and/or a cholecysto-enteric fistula which seems to be quiet complicated to ascertain for [14]. However, more validations are yet to be elucidated for the proposed grade, and for ease, this grading system could be reformed decreasing the number of grades. A recent validation study from Gyeongsang National University Hospital, Korea, concluded statistically that high PGS grades were related to frequent gangrenous cholecystitis and higher level of C-reactive protein which we could demonstrate for gangrenous cholecystitis [15]. Similarly, a study from Japan tried to stratify levels of GB inflammation using Tokyo guidelines in 2006; however, this took into account only patients who presented with acute cholecystitis and preoperative findings [16].

Limitations of the following evaluation study on usefulness of PGS include limited sample size and single institutional study. Only four surgeons classified the intraoperative first view image according to grades though maximum number of cases were rated by the corresponding author which we believe could have subjective variation if assessments were done by a greater number of surgeons and biasness do exist when interobserver variability has not been assessed. More such studies assessment and validation along with multicentre data could actually delineate the potential of the PGS grade and check its reliability and applicability globally.

## 5. Conclusion

PGS can be applied in patients undergoing laparoscopic cholecystectomy in the rural setting of a developing nation. With its application, postoperative course could be predicted and adequate counselling can be done about the possibilities of the outcome.

## Figures and Tables

**Table 1:** Parkland grading scale for cholecystitis [3].

Cholecystitis severity grade	Description of severity
1	Normal appearing gall bladder (Robin egg blue) -No adhesion present -Completely normal gall bladder.......
2	Minor adhesions at neck, otherwise normal gallbladder -Adhesion restricted to the neck or lower of the gall bladder
3	Presence of any of the following: -Hyperemia/pericholecystic fluid/adhesions to the body/distended gall bladder
4	Presence of any of the following: -Adhesion obscuring majority of gallbladder -Grade I-III with abnormal liver anatomy, intrahepatic liver, or impacted stone (Mirrizi)
5	Presence of any of the following: -Perforation/necrosis/inability to visualise the gallbladder due to adhesions

**Table 2:** Patient perioperative characteristics.

	Grade 1 (*N* = 40)	Grade 2 (*N* = 90)	Grade 3 (*N* = 26)	Grade 4 (*N* = 16)	Grade 5 (*N* = 6)
Mean age, (years) ± S.D	41.25 ± 12.7	45.47 ± 15.8	50.3 ± 16.9	45.6 ± 11.4	56.6 ± 16.9
Sex (F:M)	34 : 6	70 : 20	24 : 2	14 : 2	4 : 2
Aspartate aminotransferase (AST), (units/L) ± S.D	26.7 ± 29.4	28.1 ± 17.2	25.4 ± 9.2	43.4 ± 54.3	56 ± 10.4
Alanine aminotransferase (ALT), (units/L) ± S.D	24.6 ± 15.6	27.3 ± 20.4	22.8 ± 11.7	57 ± 88	67.7 ± 14.0
Total bilirubin, (mg/dL) ± S.D	0.87 ± 0.18	1.08 ± 0.5	0.98 ± 0.2	1.3 ± 0.6	0.9 ± 0.9
Hemoglobin, (g/dL) ± S.D	12.4 ± 1.14	12.7 ± 1.17	12.1 ± 1.07	11.6 ± 1.3	12.9 ± 1.6
Body mass index (BMI), (kg/m^2^) ± S.D	23.5 ± 4.12	24.7 ± 4.4	23.6 ± 3.9	29.7 ± 6.0	22 ± 3.4
Gallbladder wall (GBW) thickness (mm) ± S.D	3.06 ± 0.7	3.2 ± 1.3	3.8 ± 1.4	2.7 ± 0.6	5.4 ± 1.9
Pericholecystic collection present	0/40	2/88	4/22	2/14	2/4
Adhesion due to previous surgery	0	0	4	2	0

**Table 3:** Association of perioperative gall bladder grade with other surgical parameters.

	Grade 1	Grade 2	Grade 3	Grade 4	Grade 5	*p* value
Subtotal cholecystectomy	0	0	2	2	4	*p* = 0.0001 *χ*^2^ = 63.157 Df = 4
Lap converted open	0	0	2	4	6	*p* = 0.0001 *χ*^2^ = 100.9 Df = 4
Bile leak (post-op)	0	0	0	1	2	*p* = 0.0001 *χ*^2^ = 40.95 Df = 4
Pre-op WBC, (mm^3^) ± S.D	7.4 ± 1.7	7.5 ± 2.6	7.7 ± 1.8	9.5 ± 2.0	12.6 ± 1.6	*p* = 0.0001 Df = 4, Welch's ANOVA
Length of surgery, (mins) ± S.D	57.25 ± 21.45	61.67 ± 13.7	62.31 ± 10.1	67.5 ± 10.9	91.67 ± 22.06	*p* = 0.0001 Df = 4 *F* = 6.937

## Data Availability

The data used to support the findings of this study may be released upon application to the institutional review board committee at Lumbini Medical College and Teaching Hospital that can be contacted through the corresponding author.
